# Theoretical assessment design at a South African school of nursing: A multimethod qualitative exploration

**DOI:** 10.1371/journal.pone.0353215

**Published:** 2026-07-09

**Authors:** Gabieba Donough, Katlego Mthimunye, Felicity Daniels

**Affiliations:** 1 School of Nursing, Faculty of Community Health and Sciences, University of the Western Cape, Cape Town, South Africa; 2 Faculty of Health and Life Sciences, Department of Nursing, Midwifery and Health, Northumbria University, Newcastle Upon Tyne, United Kingdom; University of Hafr Al-Batin, SAUDI ARABIA

## Abstract

**Introduction:**

Theoretical assessment design is crucial in nursing education, ensuring students develop cognitive and problem-solving skills for clinical practice. However, misalignment with learning outcomes and inconsistent cognitive level distribution remain complex issues.

**Methods and findings:**

This multimethod qualitative study explored theoretical assessment design in a South African nursing school through in-depth interviews with nurse educators and a document review of moderators’ reports. Stratified purposive sampling ensured diverse representation across National Qualifications Framework Levels 5–8. Data saturation was reached after nine interviews, analysed using Creswell and Creswell’s six-step thematic framework.

The document review analysed 70 moderation reports (22 internal and 48 external) from 2015 to 2019, focusing on feedback related to final theoretical assessments. Content analysis, following Krippendorff’s framework, was used to identify themes and patterns. Findings revealed an overemphasis on lower-order cognitive skills (Bloom’s taxonomy), inconsistent question distribution, and misalignment with national qualification standards. Educators acknowledged these issues but cited time constraints, inadequate training, and institutional pressures as contributing factors. Moderation reports confirmed assessment inconsistencies, emphasising the need for better alignment with constructive alignment principles. Triangulation of data highlighted a gap between perceived best practices and actual assessment quality, suggesting assessments do not fully support higher-order cognitive skill development.

**Conclusion:**

To improve the validity and reliability of theoretical assessments, nursing programmes should prioritise training in assessment design, strengthen alignment with learning outcomes, and implement moderation strategies to address inconsistencies. These findings contribute to the broader discourse on improving assessment practices in nursing education globally.

## Introduction

Assessment design plays a critical role in nursing education, serving as a key instrument for assessing students’ theoretical knowledge and ensuring their preparedness for clinical practice [1–3]. Unlike other fields, nursing education requires assessments that not only test knowledge retention but also assess students’ ability to apply theoretical concepts in critical, patient-care scenarios, making the quality of assessment design essential for safe and effective clinical practice [4–6]. Theoretical assessments provide a structured means of measuring students’ cognitive abilities, problem-solving skills, and understanding of fundamental nursing concepts [1,2]. Beyond assessing knowledge retention, they contribute to the development of higher-order thinking skills necessary for effective clinical decision-making [7]. Thus, quality theoretical assessments are essential for bridging the gap between theory and practice, ensuring that nursing graduates possess the competencies required to provide safe and effective patient care [8].

Quality assurance of assessment design is supported through moderation processes, which are central to maintaining the integrity and standardisation of assessments in higher education [9]. Internal moderation, conducted within the institution, ensures consistency in marking and alignment with learning outcomes, while external moderation provides an independent review to verify adherence to national standards and benchmarking across institutions [10]. Together, these processes enhance the validity and reliability of assessments. In nursing education, moderation ensures that assessments align with academic standards, maintain consistency in marking, and uphold fairness in evaluation [11,12]. The moderation process identifies discrepancies in assessment design, cognitive level distribution, and question validity, ensuring compliance with national qualification frameworks and institutional policies [2,13]. This enhances the credibility and reliability of assessment outcomes, ultimately supporting the quality of nursing education. As mandated by the South African Qualifications Authority (SAQA), moderation ensures that assessments remain valid, reliable, and aligned with the National Qualifications Framework (NQF) level descriptors, thereby maintaining academic integrity across institutions [9,14].

Nursing programmes comprise both theoretical and clinical components, each playing a significant role in the education and training of future nurses. Designing assessments for these components is a key responsibility of nurse educators [15,16]. Theoretical assessments, on which this study is based, are the gathering of information to judge students’ performance against registered national standards and the programme’s learning outcomes. While this research concentrates on theoretical assessments, it acknowledges the vital role of integrating theory into practice in nursing education. As Berndtsson, Dahlborg [17] emphasise, incorporating theoretical knowledge into clinical practice is essential for developing competent nursing professionals. Although this study focuses on theoretical assessments, it acknowledges that strengthening cognitive skills through well-designed assessments can enhance students’ ability to apply knowledge in clinical settings [18].

Despite established theoretical and regulatory guidance, the design of theoretical assessments remains challenging. In higher education contexts, assessments are sometimes designed with limited alignment to national standards or cognitive complexity expectations, potentially undermining the development of critical thinking and problem-solving skills required for professional practice. Frameworks such as constructive alignment and Bloom’s taxonomy, together with guidelines provided by SAQA and the NQF, aim to address these issues, yet challenges persist. Developments in higher education appear to have influenced assessment design [19], and assessments are frequently designed at lower-order cognitive taxonomy levels [20,21]. Contributing factors include rapid technological advancement, widening access to higher education, and increasing class sizes in South Africa [8]. These changes have required adjustments in teaching and assessment practices, including the integration of new approaches and technologies to maintain educational relevance [22]. Consequently, designing high-quality assessments remains complex for educators [23].

Evidence from prior studies reinforces these concerns. Internationally, challenges in assessment design, including misalignment and over-reliance on lower-order cognitive skills, have been reported across health professions education, highlighting the global relevance of improving assessment practices [24]. Nationally, Fayilane [21] reported that final assessment papers at a South African nursing school were largely set at lower cognitive levels when analysed using the revised Bloom’s taxonomy. Similar findings have been reported in other disciplines, including Economic and Management Sciences [20], Social Studies [25], and Dental education [26], suggesting that the issue is not discipline-specific. At the institution under study, moderation reports have highlighted inconsistencies in assessment design relating to question distribution, cognitive level alignment, and adherence to best practices. Although educators often perceive their assessments as well structured, moderator feedback indicates gaps between intended practices and actual assessment quality [27]. Additional constraints, including limited time, large class sizes, and insufficient training in assessment design, may further hinder quality development of assessments, affecting their validity and reliability [28].

In response to these challenges, this study draws on theoretical frameworks and national regulatory guidelines to explore how assessment design practices are understood and enacted in a nursing education context. The study aimed to explore and describe nurse educators’ experiences of designing theoretical assessments, and to review moderation reports on final theoretical assessments within a Bachelor of Nursing programme at a South African School of Nursing. By identifying challenges and areas of good practice, the study sought to inform the understanding of assessment design and contribute evidence that may support ongoing improvement of assessment practices.

### Theoretical frameworks

The design of theoretical assessments in nursing education is guided by well-established theoretical frameworks that ensure alignment with intended learning outcomes and cognitive development goals. Two key frameworks underpin this study: Biggs’ (1996) theory of constructive alignment and Bloom’s taxonomy of educational objectives (1956). These frameworks were chosen for their complementary strengths in guiding assessment design and their relevance to addressing the challenges identified in the study.

Constructive alignment emphasises the coherence between learning outcomes, teaching activities, and assessment tasks [12]. This approach ensures that assessments accurately measure the competencies they are designed to assess, thereby promoting meaningful learning. By aligning assessment tasks with clearly defined learning outcomes, this study seeks to determine whether assessments not only test students’ knowledge but also support their broader learning journey.

Bloom’s taxonomy provides a hierarchical model for categorising learning outcomes based on cognitive complexity. It classifies educational goals into six levels, ranging from lower-order thinking skills (e.g., knowledge and comprehension) to higher-order skills (e.g., analysis, evaluation, and creation) [29]. The revised version of Bloom’s taxonomy [30] highlights the importance of designing assessments that move beyond factual recall to foster critical thinking and problem-solving skills. This taxonomy was instrumental in analysing the cognitive demands of assessment tasks, assessing whether they were designed to cultivate higher-order thinking or remained at basic recall levels [31].

Although Bloom’s taxonomy remains one of the most widely used frameworks for assessment design, it has been subject to critique. Scholars have argued that the taxonomy presents cognitive development as a linear and hierarchical process, whereas learning is often iterative, recursive, and context dependent [32,33]. Critics have also questioned whether the revised taxonomy sufficiently addresses these limitations, particularly the positioning of “create” as the highest cognitive level [34]. Furthermore, the use of action verbs alone has been criticised as an unreliable indicator of cognitive complexity, as the overall structure, context, and intent of an assessment task ultimately determine its cognitive demand [35,36]. In health professions education, where clinical reasoning requires the integration of knowledge, judgement, and contextual decision-making, cognitive processes may not always align neatly with hierarchical categories [37]. Consequently, this study used Bloom’s taxonomy as a guiding analytical framework while interpreting cognitive complexity in relation to the overall design and intent of assessment tasks rather than verb choice alone.

Together, these frameworks serve as complementary tools in this study. Constructive alignment provides the overarching structure to ensure coherence between assessments and desired learning outcomes, while Bloom’s taxonomy offers a detailed lens to examine the cognitive levels targeted by assessment questions. These frameworks ensured that the study’s findings and recommendations are grounded in established pedagogical principles, contributing to improved assessment practices in nursing education [38].

### National Qualifications Framework and SAQA alignment

In South Africa, assessment design is regulated by SAQA and structured according to the NQF [2]. The NQF is a system that standardises educational qualifications to ensure consistency, quality, and alignment with international standards [14]. For instance, the Bachelor of Nursing degree is typically placed at NQF Level 8, reflecting its academic rigor and expected level of competence. This framework supports alignment between qualifications, learning outcomes, labour market needs, and ensuring graduates meet both national and international expectations.

SAQA provides policies and guidelines that establish principles of assessment, ensuring validity, reliability, consistency, and fairness [2]. The NQF consists of 10 levels, with level descriptors outlining the expected learning achievements and cognitive complexity at each stage [39]. For the Bachelor of Nursing degree, this requires that assessments are appropriately aligned with Level 8 expectations and support the progressive development of students’ competencies across the programme [2].

Higher Education Institutions (HEIs) are responsible for ensuring that theoretical assessments are aligned with NQF level descriptors and corresponding learning outcomes [2,40]. Moderation processes further support this alignment by identifying inconsistencies in assessment design and ensuring adherence to national standards and institutional policies [41].

## Methods

This study employed a multimethod qualitative research approach, consisting of two complementary studies using in-depth interviews and a document review. The aim was to explore nurse educators’ experiences and moderators’ feedback on the design of theoretical assessments in a school of nursing. A qualitative lens allowed for a rich understanding of subjective experiences and nuanced perspectives within the context of a school of nursing in South Africa, while triangulation of findings from the two studies ensured a comprehensive analysis of the phenomena under investigation [42].

### Research setting

The study was conducted at a nursing school within a HEI in South Africa. The institution offers both undergraduate and postgraduate nursing programmes, providing an ideal setting for exploring assessment practices due to its commitment to preparing competent nursing professionals. This study focused on the undergraduate Bachelor of Nursing programmes, specifically the 4-year Mainstream Curriculum and the 5-year Extended Curriculum Programmes (ECP).

#### Study 1: Exploratory-descriptive study.

This study employed in-depth interviews with nurse educators teaching in the undergraduate Bachelor of Nursing programme to explore their experiences in designing theoretical assessments. The population comprised 22 nurse educators, with inclusion criteria requiring participants to have taught and designed assessments in either the 4-year Mainstream Curriculum or the 5-year Extended Curriculum undergraduate programme. A stratified purposive sampling strategy was used [43], selecting participants who taught across NQF Levels 5–8 to capture a broad range of assessment experiences, from foundational level (Level 5 – First year) to advanced level (Level 8 – Final year). This approach ensured representation of diverse perspectives on assessment design across different undergraduate academic levels. The sample size was not predetermined, as qualitative studies prioritise informational adequacy rather than numerical targets [44]. Interviews continued until thematic sufficiency was reached, indicated by repetition of patterns and no substantial emergence of new conceptual insights after nine interviews. While data saturation remains debated in qualitative research, the sample provided sufficient depth and variation across NQF levels to support the exploratory-descriptive aims of this study [44]. Interview discussions were confined to experiences related to undergraduate theoretical assessment design to ensure methodological alignment with Study 2, which examined moderation documentation exclusively from undergraduate programme assessments.

An in-depth semi-structured interview guide was developed, starting with the open-ended question, *“What are your experiences of assessment design?”* Probing questions were refined after a pre-test interview in April 2021, and after receiving ethical clearance, to ensure clarity and comprehensiveness. The researcher was a staff member at the institution under study. To mitigate potential bias, bracketing was applied, and an independent research assistant conducted the interviews to enhance objectivity [43]. This approach encouraged openness, enhancing data validity [45].

Participants provided both written and verbal informed consent for participation and audio recording. Prior to each interview, the interviewer explained the aim of the study again, and participants were asked to reconfirm their permission for the interview and the recording. This verbal confirmation was documented as part of the audio recording itself, ensuring that consent was informed, witnessed, and properly recorded. Permission for the interviews was secured before the pre-test and main data collection. Due to COVID-19 restrictions at the time of data collection, all interviews were conducted online via Google Meet between May and November 2021 [43]. Each interview lasted 40–60 minutes, was audio-recorded with consent, and conducted sequentially with participants teaching in the NQF Level 5 (ECP/Year 1) to Level 8 (Year 4), repeating the cycle until data saturation was achieved [44]. Data were transcribed verbatim and analysed using ATLAS.ti 9.0 software [46]. Braun and Clarke’s six-step framework for thematic analysis was applied [44]:

1. Familiarising with the data through repeated transcript readings;
2. Generating initial codes;
3. Organising codes into potential themes;
4. Reviewing themes for alignment with the data;
5. Defining and naming themes;
6. Producing a report capturing key findings.

Trustworthiness was ensured through credibility (member checking), transferability (thick descriptions), dependability (audit trails), and confirmability (multiple researchers in analysis) [47]. The researcher also practiced bracketing and reflexivity to minimise biases, ensuring findings were data-driven.

#### Study 2: Document review of moderator’s reports.

This study involved a document review of internal and external moderators’ reports examining feedback on the design of final assessments in the Bachelor of Nursing programme. The research question guiding the study was: *“What is reported by internal and external moderators on the design of final assessments in the programme?”* The review focused on reports from the undergraduate programme (the R425 programme) across all year levels between 2015 and 2019. This timeframe was selected because it aligned with the final intake of students for the R425 programme, which was phased out nationally in 2019 [48], as a new undergraduate nursing programme phased it in 2020. The period was also chosen based on anecdotal reports from the Deputy Vice-Chancellor, which emphasised the need to evaluate assessment design over the preceding five years. This review provided insights into assessment design practices informing interpretation of the findings. A total of 150 internal and external moderation reports for 18 nursing modules were obtained in October 2020 in both electronic and hard copy formats. Inclusion criteria required reports to be complete, pertain to final theoretical assessments, and feedback on nursing modules’ assessments. Reports on non-nursing modules, postgraduate modules and clinical modules were excluded. Module names and moderator identities were anonymised to maintain confidentiality. From the 150 available reports, 70 (22 internal and 48 external reports) were selected using purposive sampling to ensure coverage of theoretical assessments across different years and academic levels. This sample ensured thematic saturation in document analysis and representation of diverse assessment practices. A data extraction tool, developed using the moderation report template, was validated for face and content validity by research supervisors. This tool ensured all relevant data were collected systematically. Content analysis, following Krippendorff’s five-step framework, was used to analyse the moderators’ reports [49]:

1. Identifying relevant content within the reports.
2. Sampling ensured that only reports meeting the inclusion criteria were selected.
3. Coding categorised data into meaningful units.
4. Synthesising the data into themes.
5. Drawing conclusions based on the extracted data.

An iterative process was followed, allowing findings from the data extraction process to refine subsequent analyses [43]. This approach ensured a thorough understanding of the moderators’ feedback and its implications for assessment design. The systematic and iterative nature of the analysis provided robust insights into the strengths and areas for improvement in the design of final assessments during the specified period.

### Data synthesis and triangulation

A multimethod approach, combining in-depth interviews and document reviews, was used to enhance the richness and depth of the findings. This approach provided multiple perspectives, enabling a more comprehensive exploration of theoretical assessment design [50]. Triangulation of the findings from the two studies further strengthened the analysis by integrating data sources, enhancing the reliability and depth of the insights [51,52]. Data synthesis involved the combining and integrating of findings from the two studies to identify common themes, patterns, and insights [53]. Triangulation involved cross-verifying data from multiple methods to ensure consistency and validity, providing a more holistic understanding of the research problem [51,52]. In this study, the findings from the in-depth interviews (Study 1) and the document review (Study 2) were triangulated and synthesised. The document review provided structured, macro-level insights from internal and external moderators, highlighting formal feedback on assessment practices. In contrast, the in-depth interviews offered detailed, nuanced accounts of nurse educators’ experiences, providing a micro-level perspective. For instance, while nurse educators expressed confidence in the cognitive level distribution of their assessments, most of the moderation reports indicated a dominance of lower-order recall-based questions, highlighting a misalignment between perceived and actual assessment quality. This comparison of qualitative findings provided a nuanced understanding of assessment challenges, validating the study’s results. By comparing and contrasting the findings from both datasets, common themes and categories were identified, and insights were integrated to form a cohesive understanding of theoretical assessment design. This process revealed key challenges, successes, and opportunities for improving assessment practices. The triangulated analysis ensured that the resulting themes were robust and well-rounded, reflecting a comprehensive view of assessment design within the study context. This integrated approach not only validated the findings but also provided a richer, more nuanced understanding of the topic.

### Ethical considerations

The study received ethics clearance from the Human Social Sciences Research Ethics Committee at the institution where the research was conducted (Reference number: HS20/8/19). Additional permission was granted by the registrar and the director of the school of nursing. To protect participants’ privacy and confidentiality, several measures were implemented. Nurse educators participating in the interviews provided informed consent for both participation and audio recording. They were assured that participation was voluntary, they could withdraw at any time without consequences, and their responses would remain confidential [43]. All identifying information, such as names and affiliations, was removed during transcription. Similarly, in the document review, module names, moderator names, and other identifiable details were anonymised to protect individuals and maintain confidentiality. Data security was ensured by storing electronic data, including recordings and transcripts, on password-protected, encrypted devices accessible only to the research team. In line with institutional and ethical guidelines, data will be retained for five years before being securely destroyed. These measures upheld ethical standards, safeguarding the privacy and confidentiality of all participants and data throughout the research process.

## Findings and discussion

This section presents the findings from Study 1 (in-depth interviews with nurse educators) and Study 2 (document review of moderation reports), as well as the triangulated findings that integrate insights from both data sources. The findings are interpreted using the theoretical frameworks of Biggs constructive alignment and Bloom’s taxonomy, which provided a structured lens to examine the alignment of assessments with learning outcomes and the cognitive levels targeted by assessment tasks.

Constructive alignment guided the analysis by identifying areas where theoretical assessments were effectively aligned, or misaligned, with the intended learning outcomes. Simultaneously, Bloom’s taxonomy allowed for a detailed examination of whether assessment tasks promoted higher-order cognitive skills such as analysis, evaluation, and creation, or remained focused on lower-order skills like recall and comprehension.

Table 1 below summarises the key findings from Study 1, Study 2, and the triangulated findings, offering a comprehensive understanding of the challenges, successes, and opportunities in theoretical assessment design at the nursing school. This synthesis highlights how the perspectives of nurse educators and moderators complement one another, contributing to a holistic view of assessment practices. By integrating these insights, the study provides a nuanced understanding of how assessment design can be improved to better align with learning outcomes and foster higher-order thinking skills, ultimately enhancing the quality of nursing education.

**Table 1:** Integrated findings of in-depth interviews and document review.

Theoretical Framework	Integrated themes	Integrated categories
**Biggs Constructive Alignment**	Theme 1: Assessments must be constructively aligned to the NQF and scaffolded according to Bloom’s taxonomy	Category 1a: Constructively aligned assessments Category 1b: Use of Bloom’s taxonomy to ensure high standards and scaffolding Category 1c: NQF levels should guide assessment design
**Bloom’s Taxonomy**	Theme 2: Assessment must be based on assessment principles and policies	Category 2a: Moderation to quality assure assessment design Category 2b: Assessment design must be in accordance with internal and external assessment policies Category 2c: Adherence to the principles of assessment
	Theme 3: Assessment structure, use of language, and technical presentation	Category 3a: Assessment structure must be valid Category 3b: Assessments must be grammatically and technically sound
	Theme 4: Constraints for designing quality assessments	Category 4a: Challenges and time constraints in assessment design Category 4b: Language barriers in assessment design
	Theme 5: Contributions to the successful design of assessments	Category 5a: Being innovative and creative to design assessments Category 5b: Experience and qualification of nurse educator Category 5c: Professional development, mentorship, and collaborative support

### Theme 1: Assessments must be constructively aligned to the NQF and scaffolded according to Bloom’s taxonomy

This theme highlights the importance of constructive alignment, a concept proposed by Biggs [41], and the NQF. It highlights the use of Bloom’s taxonomy to scaffold assessments, ensuring a progression from lower-order to higher-order thinking skills. Educators emphasised the need align assessments and to design assessments that promote critical, reflective, and analytical thinking, essential for professional nursing practice [54]. However, the moderation reports revealed a predominance of lower-order thinking skill questions in the assessments.

#### Category 1a: Constructively aligned assessments.

The participants reported that while designing assessments, the learning outcomes and related assessment criteria must be considered. They mentioned:

“In the design, you still have to… align your assessment with… the learning outcomes.” (P6).

“[to design an assessment] you need to look at… all modules. All modules should have assessment criteria in place” (P1).

Learning outcomes are a critical component of curriculum design, and assessments must align with both the NQF and these outcomes [2,55]. According to Kintu, Zhu [56], the NQF level and learning outcomes are key considerations in assessment design. This implies that assessments should increase in complexity as students’ progress through higher NQF levels. However, moderation reports identified gaps in this alignment:

“Each paper lacked assessment of all specific outcomes for this module” (2^nd^-year internal moderator 1).

“Not sure what year level this module is presented at [lack of alignment]” (4^th^-year external moderator 1).

This misalignment suggests that assessments did not comprehensively cover module outcomes, undermining their validity. Educators must ensure that assessments reflect the required competencies and outcomes, as highlighted by Mariam, Sapriati [57]. Disparities between learning outcomes, teaching, and assessments can hinder constructive alignment [58–60]. Recognising the significance of learning outcomes can guide educators in designing assessments that accurately reflect these outcomes and adhere to established assessment principles.

#### Category 1b: Use of Bloom’s taxonomy to ensure high standards and scaffolding.

Nurse educators consistently referenced Bloom’s taxonomy as a foundational tool for designing assessments. Participants highlighted its role in structuring questions and ensuring high academic standards:

“I look at things like Bloom’s taxonomy and how I use my verbs when I formulate my exam papers” (P1).

“Yeah, it’s the Bible [Bloom’s taxonomy]” (P7).

These comments illustrate the educators’ recognition of Bloom’s taxonomy as a valuable tool for structuring assessments. Aligning with the work of Kumara, Brahmana [61], Bloom’s taxonomy provides clear guidance for achieving high standards in assessments. This study operated on the widely adopted assumption that taxonomies of learning, particularly Bloom’s taxonomy, provide a useful framework for structuring cognitive progression in assessment design. However, this position is not universally uncontested. Scholars caution that cognitive level cannot be inferred solely from action verbs, as the overall structure, context, and cognitive demand of a question ultimately determine complexity [62]. Acknowledging this perspective situates the interpretation of findings within ongoing scholarly debate. Educators also demonstrated an understanding of scaffolding by designing assessments that progressively build students’ cognitive abilities. For example:

“The difference comes in between the first and the fourth year… we utilise Bloom’s taxonomy” (P4).

“…your scenarios based on knowledge or recall and maybe, for them to be able to understand and then you move on to a more complex… it’s almost like you are… it is scaffolding…” (P6).

These insights align with scaffolding principles, where assessments are designed to gradually increase in complexity, allowing students to develop from novices to experts by fostering critical thinking and clinical reasoning [63]. This scaffolding approach ensures assessments transition from lower-order skills, such as recall and comprehension, to higher-order skills, such as analysis, evaluation, and creation [64]. Therefore, by starting with lower-order thinking skills, an educator implementing Bloom’s taxonomy aims to develop students from being a novice to being an expert, by providing opportunities for critical thinking and clinical reasoning [65]. Participants also linked this progression to NQF levels, emphasising the importance of aligning assessments with students’ cognitive development:

“Pitching your questions at the correct NQF level for first years… you will mainly formulate your questions based on knowledge or recall, then move to more complex tasks, it is scaffolding” (P6).

“As you move to fourth years at NQF Level 8, you will have more questions requiring synthesis, analysis, and evaluation” (P7).

“When we utilise Bloom’s taxonomy, we will utilise the higher-order thinking skills” (P4).

These findings confirm that educators systematically increase the complexity of assessments in line with NQF levels, fostering students’ independence and preparing them for professional practice. This means encouraging students to move beyond rote memorisation and recall, enabling them to apply fundamental knowledge to higher-order thinking skills and practical scenarios [63]. By progressing through the cognitive levels of Bloom’s taxonomy, students develop the ability to critically analyse, synthesise, and evaluate information independently, equipping them with the skills necessary for professional practice.

Nurse educators play a vital role in preparing students to become competent professionals by designing assessments aligned with the appropriate level of complexity. Moreover, according to Billings and Halstead [66], Chinembiri [67], and Govindasamy and Kwe [63], assessments in higher education must promote critical, reflective, and analytical thinking, essential for nursing practice. Graduates from the programme will be expected to apply their knowledge effectively in real-world clinical settings where they work, where quick and informed decision-making is essential, a sentiment shared by Bell and Brysiewicz [68]. Kumara, Brahmana [61], Watson [69], and Zaidi, Grob [64] concur that Bloom’s taxonomy serves as a framework for stimulating this type of learning, encouraging educators to design assessments that inspire deeper engagement and understanding.

The moderation reports, however, revealed significant gaps in the cognitive levels of assessment questions:

“Level of questions is primarily based on understanding” (3^rd^-year external moderator 1). “The formulation of MCQs should be at higher levels” (3^rd^-year external moderator 2).

A study conducted by Mirbahai and Adie [70] at the University of Warwick in the United Kingdom sheds light on the challenges associated with multiple-choice questions (MCQs) designed at lower cognitive levels. The research identified item-writing flaws in the design and coverage of MCQ questions, raising concerns about their validity and effectiveness in assessing students’ understanding. Mirbahai and Adie [70] suggest that while MCQs have the potential to assess cognitive domains across various levels, such as promoting problem-solving abilities that require students to apply their knowledge to clinical case scenarios, proper training for educators in assessment design is essential to ensure the quality and reliability of assessments. Moderators raised concerns about the prevalence of lower-order thinking questions, even in senior-year assessments. For instance, a fourth-year moderator commented:

“Some suggestions are given in specific papers to restructure questions to stimulate higher cognitive thinking” (4^th^-year internal moderator 2).

“I would like to see less knowledge questions in the next paper” (4^th^-year external moderator 3).

These observations suggest a misalignment between the intended cognitive levels of assessments and their actual design, particularly at higher NQF levels. This implies that it could limit students’ opportunities for deep learning, critical thinking, and skill development which are essential for success in higher education and professional practice in the workforce. Sakzad, Paul [71] conducted a qualitative study highlighting that the complexity level of assessment questions significantly influences students’ performance. Similarly, dos Reis, Swanepoel [20] emphasised the importance of question complexity in determining students’ grades, indicating that assessments pitched at lower cognitive levels may affect the number of students achieving higher distinctions and pass rates. This concern resonates experience in the school of nursing. Failure to align assessment complexity with cognitive level criteria could raise doubts about the validity of students’ achievements. Therefore, ensuring assessments meet the required cognitive complexity levels is crucial for maintaining the integrity and validity of student outcomes in nursing education programmes.

#### Category 1c: NQF levels should guide assessment design.

The NQF serves as a guiding framework for designing assessments that align with students’ academic progression and cognitive development [2]. Participants emphasised the importance of tailoring assessments to reflect the appropriate NQF level:

“If you use your NQF level, you will not go wrong… the first thing is I will print my NQF level…” (P2).

“Your NQF level…you also need to take into consideration when designing assessment;” (P8).

“When we set the paper, you consider the level of the student… you cannot set a question that can be answered by first-year and fourth-year students alike” (P5).

This alignment ensures assessments are pedagogically sound, challenging, and accessible, supporting students’ learning and preparation for professional practice. A participant teaching at a lower academic level illustrated this by stating:

“Pedagogically you have failed if the students were not able to understand because you pitched it too high that it was above their reasoning capacity” (P2).

These findings demonstrate that educators viewed the NQF as a structural benchmark for determining assessment complexity and progression across academic levels. Unlike Bloom’s taxonomy, which guided cognitive formulation of questions, the NQF provided a contextual and regulatory framework ensuring level-appropriate expectations within the national qualification structure.

Moderation reports provided additional confirmation that assessment design was expected to align with programme level descriptors. Moderators frequently evaluated whether questions were pitched at an appropriate level of complexity and highlighted deviations from expected standards. For example, comments such as:

“Level of questions is primarily based on understanding” (3^rd^-year external moderator 1)

“MCQ’s can be formulated at a higher level of knowledge” (3^rd^-year external moderator 2)

These comments indicate concerns that assessments were not always aligned with the expected academic level. In several instances, moderators also requested curriculum documentation to determine whether the level of learning measured was appropriate, reflecting the role of national level descriptors in judging assessment quality. These observations reinforce the function of the NQF as an implicit benchmarking mechanism within moderation processes, ensuring assessments reflect appropriate complexity and progression across programme levels.

### Theme 2: Assessment must be based on assessment principles and policies

Assessment policies play a crucial role in ensuring the integrity, validity, and fairness of student evaluations. According to Yan and Brown [72], a well-founded assessment policy that is embedded in governance structures and recognises assessment as a core component of teaching and learning enhances academic standards. This theme emerged from participants’ discussions on assessment principles, institutional and external policies, and the role of moderation in ensuring quality assurance. However, a notable gap in the moderation reports was the absence of explicit references to assessment principles and policies, highlighting a potential misalignment between policy expectations and actual practices.

#### Category 2a: Moderation to quality assure assessment design.

Moderation serves as a quality assurance mechanism that enhances the credibility and validity of assessments [11]. In the school of nursing, all assessments undergo a moderation process as part of institutional quality assurance procedures. Participants shared their experiences with moderation feedback:

“We have a moderator… who goes through the question to check the grammar, the content, alignment of the questions to the learning outcomes...” (P5).

“…the comments were basically around … [referring to the appropriateness of the assessment papers for the NQF level] and just recommendations on how I can improve the papers” (P3).

These comments indicate that moderation practices are in place to ensure alignment with assessment principles and policies. Research suggests that effective moderation enhances assessment reliability and promotes consistency across academic programmes [9,19]. However, research has also highlighted inconsistencies in moderation practices across higher education institutions (HEIs) in South Africa, with some institutions failing to fully integrate moderation as a crucial quality assurance mechanism [9,73]. Furthermore, the effectiveness of moderation depends on the extent to which feedback is utilised to improve assessment design. Ajibade and Hayes [4], Boud and Bearman [74] argues that constructive feedback through moderation fosters continuous enhancement in assessment practices, ensuring that assessments remain rigorous and pedagogically sound. The absence of assessment principles and policy references in moderation reports suggests a gap in ensuring systematic alignment between policy directives and actual assessment practices, warranting further institutional intervention.

#### Category 2b: Assessment design must be in accordance with internal and external assessment policies.

Educators must adhere to both institutional and regulatory policies when designing assessments. Several participants acknowledged the role of institutional and national policies in guiding assessment practices:

“…the school of nursing operating procedures…or the procedure guide, …give some guidance…in terms of how you would go around setting [assessments]” (P9).

“We also belong to the South African Nursing Council, which is our quality assurance body. Yes, that’s correct it’s, SANC. And they set our nursing-specific standards” (P8).

The South African Nursing Council (SANC) assumes responsibility for setting and maintaining educational and professional standards in nursing within the country [75]. As an external regulatory body, SANC oversees the quality of nursing education, ensuring that assessments align with national accreditation requirements [58]. Additionally, the SAQA [2] provides guidelines that regulate assessment standards across various qualification levels.

Both internal and external policies serve as fundamental frameworks for designing assessments that are valid, reliable, and fair [13,76–79]. However, research by Villarroel, Bruna [80] suggests that while policies exist, their implementation often varies across institutions due to differences in educator interpretations and institutional support structures. This reinforces the need for ongoing professional development to enhance educators’ understanding of policy-driven assessment practices.

#### Category 2c: Adherence to the principles of assessment.

To maintain integrity and fairness in assessments, educators must adhere to key assessment principles, including validity, reliability, fairness, and flexibility [80–83]]. Participants highlighted their awareness of these principles:

“Our assessment policy is very clear on… how the assessments should look like. What are the principles… it should be fair; students shouldn’t be tested about something that they haven’t… been exposed to” (P8).

“…just your normal pedagogic principles like fairness in questions. Not setting unnecessarily difficult questions” (P2).

“I said, so now why are you asking them a different question? If the student was never taught this” (P9).

These responses suggest that participants recognise the importance of fairness and validity in assessment design. Validity, in particular, ensures that assessments accurately measure what they are intended to assess [81,83]. Biggs [84] emphasise that validity is closely tied to constructive alignment, assessments must align with intended learning outcomes and teaching activities to be considered valid.

Reliability is another critical principle, ensuring consistency in assessment results across different contexts [47]. Research has shown that unclear assessment criteria and subjective marking often undermine reliability [13,47,85]. Flexibility in assessment design is also essential, allowing for diverse assessment methods that accommodate different learning styles [86].

Despite participants’ awareness of these principles, there is a need for structured training in assessment literacy. Studies indicate that educators often apply assessment principles inconsistently due to limited exposure to formal assessment training [3,80,87]. Enhancing educators’ assessment literacy through targeted workshops and professional development programmes can improve the quality and fairness of assessments in nursing education.

### Theme 3: Assessment structure, use of language, and technical presentation

A well-structured assessment is essential to ensure validity, fairness, and clarity in student assessment. Proper mark allocation, coherent language use, and technical soundness all contribute to an effective assessment process [88–90]. This theme emerged from educators’ reflections on assessment structure, moderation reports, and their experiences with assessment design.

#### Category 3a: Assessment structure must be valid.

Assessment validity relies on careful planning, appropriate question types, and precise mark allocation [19]. Participants emphasised:

“You need to have a set picture or frame in your mind of exactly how you want to have the end product. And then the outcome? …you look at what it is that you want to assess?” (P6).

“Your mark allocation must reflect the importance of the assessment and the effort that the student put into that assessment” (P6).

Moderation reports highlighted inconsistencies:

“There are no allocated marks for these questions” (ECP & 1^st^-year internal moderator 1).

“If you really want students to discuss this issue, 4 marks are not enough” (3^rd^-year external moderator 3).

“Concerned about the time frame 2 hours for 50 marks” (2^nd^-year external moderator 3).

The findings emphasise the critical importance of designing assessments with a clear structure, appropriate question types, and precise mark allocation to ensure validity. Participants highlighted the need for a well-defined framework that aligns assessment tasks with intended outcomes and reflects the effort required from students. However, moderation reports revealed inconsistencies in mark allocation, timeframes, and the depth of questions, which could compromise the validity and fairness of assessments. Addressing these issues through careful planning and alignment with assessment principles is essential to ensure that assessments accurately measure student learning and uphold academic standards.

#### Category 3b: Assessments must be grammatically and technically sound.

Grammatical and technical errors can lead to confusion and misinterpretation [91]. Educators noted:

“… you will check the grammar; you will check the formatting” (P5).

Moderation reports however identified issues:

“Grammar and spelling need attention” (3^rd^-year external moderator 4).

“Sentence constructions and grammar for scenarios required moderation” (2^nd^-year internal moderator 2).

Poor grammar usage appears to be a major concern in assessments, as it can negatively impact students’ understanding of questions and responses. El-gohary [92] conducted a review at Taibah University, Egypt, analysing 36 multiple-choice question assessments and found that 45% of them contained improper grammar. This highlights the importance of training assessors to ensure grammatical correctness in assessments. Similarly, Purpura [93] emphasised the significance of correct grammar in assessments based on a qualitative study conducted at Columbia University, USA. Ensuring grammatically and technically sound assessments is crucial for maintaining assessment integrity and effectiveness [94]. Institutions should provide training and clear guidelines to assist educators in designing assessments that meet these standards. A structured, grammatically accurate, and technically sound assessment enhances its reliability and fairness. Addressing inconsistencies in mark allocation and improving assessment literacy through training can significantly enhance assessment quality.

### Theme 4: Constraints for designing quality assessments

Providing clear guidance to nurse educators on the design of theoretical assessments is crucial for ensuring quality. However, various constraints impact their ability to develop effective assessments [27]. Participants in this study reflected on the challenges they faced in assessment design, with time constraints emerging as a dominant factor.

#### Category 4a: Challenges and time constraints in assessment design.

The majority of participants described assessment design as a difficult and time intensive process:

“It wasn’t easy. It wasn’t easy” (P1). “You know to set up an assessment is not easy” (P2). “It was a bit daunting and overwhelming” (P3). “So, setting up papers can be very challenging” (P4).

The majority of participants explained that it was not an easy task to design assessments. Assessment is very useful as it supports student learning, however, it is not always easy to design [28,72]. When probed why it was not easy, the following came to light: “I wasn’t guided on that. I had no direction” (P3).

The comment above confirms a lack of guidance and support for designing assessments. In addition, participants described the workplace as having a distinct but perplexing ambience during the final assessment period:

“There’s this atmosphere, this environment that you must not sleep, everything must be now because the paper must go in” (P4).

The above comment highlights that educators work under pressure to meet deadlines during the final assessment period due to time constraints. Massoudi and Hamdi [95] argue that workplace environments, including time pressures, significantly impact productivity. This aligns with the challenges reported by participants in designing assessments. Time constraints emerge as a notable challenge, impacting educators’ ability to design assessments effectively.

Time constraints were frequently mentioned as a key challenge:

“…the one thing that is sometimes difficult, is that you’re always under such a lot of pressure in setting your papers, you know, … terms of time” (P8).

“…because assessment is a challenge… in the university… I’m also part of the… assessment committee, and this is not peculiar to Community and Health Sciences only” (P9).

These challenges often led to compromises in the quality of assessment design. Moderation reports echoed these concerns and included suggestions for improvement:

“There are some suggestions I made in terms of clarity of questions” (ECP & 1^st^-year internal moderator 2).

“Check questions… and correct just to guide the student and to make more understandable” (3^rd^-year internal moderator 5).

Educators face challenges in designing assessments as evidenced by moderation reports and participant feedback. Research supports these findings, indicating that a busy academic calendar and workplace pressures significantly restrict educators’ ability to dedicate sufficient time to quality assessment design [96,97]. Additionally, workplace environments often create pressure to meet deadlines [95], further complicating assessment design. While time constraints were identified, the gap between conceptual understanding and enactment likely reflects broader systemic factors including limited formal training in assessment literacy, reliance on heuristic verb-based interpretations of Bloom’s taxonomy, and institutional workload pressures. Previous scholarship indicates that educators often possess theoretical awareness but lack practical support structures for translating this knowledge into complex item construction, particularly at higher cognitive levels.

#### Category 4b: Language barriers in assessment design.

Some participants reported language barriers as a challenge that prevented them from designing quality assessments:

“Language plays a big role. My home language is not English. I have not trained in English; I have a better understanding of many things in my own mother tongue than in English… you also need to look at will the student understand it” (P7).

“We are not writers... you would find the language unclear. I’m setting a paper in English. And when the papers written it, it’s just unclear” (P9).

Moderation reports further highlighted these issues, identifying specific problems with language clarity and structure:

“Sentence constructions and grammar for scenarios required moderation” (2^nd^-year internal moderator 3).

“Typing errors; questions that need rewording/ reconstruction” (3^rd^-year internal moderator 6).

English is the medium of instruction at the university and the school of nursing included in the study, which places additional pressure on educators to design assessments in a language that may not be their first language. Participants emphasised that limited proficiency in English hindered their ability to write clear and precise assessment questions, which could affect students’ understanding and performance. Lobão, Coelho [98] argue that individuals with limited proficiency in English may struggle with technical terminology and complex concepts, further complicating the assessment design process. These language barriers, combined with limited training, guidance, and time constraints, contribute to the broader challenges in assessment design. Addressing these issues requires structured support, such as professional development programmes focused on academic writing and assessment design, as well as access to language resources and editing assistance.

### Theme 5: Contributions to the successful design of assessments

The nurse educators reflected on their qualifications and expertise, recognising their significance in effective assessment design. They emphasised the importance of capacity building through continuous professional development.

#### Category 5a: Being innovative and creative to design assessments.

Participants highlighted the need for innovation in assessment design:

“You need to continuously adapt your assessment methods because students are changing… what worked before does not always work now” (P9).

“You really need to always look at new resources, you really need to be innovative” (P8). “You really need to upskill yourself or you need to keep yourself abreast of technology. So, attend many of these sessions, practice, speak to colleagues” (P9).

Enhancing one’s skills through continuous learning is essential for staying current and fostering creativity in assessment design. Creativity and authenticity are closely intertwined, as highlighted by Bryan and Clegg [99], with authentic and creative assessments promoting student engagement and learning [57,71]. Professional development through training workshops fosters innovation, allowing educators to improve their assessment practices.

#### Category 5b: Experience and qualification of nurse educator.

Participants emphasised how experience and qualifications contribute to effective assessment design:

“With more years of teaching, you learn how to set better questions… you start seeing what works and what doesn’t” (P7).

“It’s my field. I’m the expert in my field. And I’m the one who taught the students… So, I know this question is okay for them” (P2).

“You take your own experience, you take the theory, you put that together... it will be very difficult for you… if you don’t have practical experience.” (P7).

These insights highlight how subject expertise and practical experience enable educators to design assessments that align with learning outcomes and teaching and learning activities. However, moderation reports identified issues that may reflect a lack of experience or expertise among some educators:

“This paper does not reflect well balanced content covered for semester 1.” (ECP & 1^st^-year internal moderator 3).

“Some questions have two questions in one.” (3rd-year external moderator 7).

Such issues suggest that novice educators, in particular, may struggle with designing balanced and coherent assessments. Research supports the importance of experience and qualifications in assessment design. For instance, Looney, Cumming [100] emphasise that proficiency in assessment design develops gradually over time, underlining the need for ongoing professional development. Similarly, Norton, Floyd [90] argue that practical experience and subject expertise are crucial for designing effective assessments. Novice educators, who may lack these competencies, require structured training and mentorship to enhance their assessment design skills. As highlighted by El-gohary [92], targeted professional development programmes can help bridge this gap, enabling educators to design assessments that are both rigorous and aligned with learning outcomes.

#### Category 5c: Professional development, mentorship, and collaborative support.

Participants emphasised the role of self-empowerment in assessment design:

“You can book a training… so that you can empower yourself” (P5).

“During the induction period… staff members should be guided and assisted in setting assessments” (P6).

“When you are new, you rely a lot on colleagues to guide you in setting papers… you don’t just know how to do it” (P6).

“We often discuss assessments as a team… it helps to get input from others before finalising” (P8).

Mentoring, induction programmes, and continuous training enhance assessment literacy and design skills [3,101]. Empowering educators through professional development ensures sustainable improvements in assessment quality.

## Conclusion

This study explored assessment design in nursing education through in-depth interviews with nurse educators and a document review of moderators’ reports, offering insights into the challenges and practices in designing theoretical assessments. While educators demonstrated an understanding of best practices, significant barriers, such as time constraints, large class sizes, and limited training hindered implementation, resulting in assessments that often prioritised lower-order cognitive skills. These findings highlight the need for assessments that scaffold cognitive progression, align with constructive alignment principles, and are supported by institutional structures.

While Bloom’s taxonomy provided a useful framework for analysing assessment complexity, the findings were interpreted critically, recognising that cognitive demand is influenced by the overall structure and intent of assessment tasks rather than action verbs alone. This approach strengthened the interpretation of assessment quality within the context of nursing education.

The findings further suggest that strengthening moderation practices, peer engagement, and collaborative approaches to assessment design may support improved alignment between intended and enacted practices. In addition, professional development and workload considerations appear important in enabling educators to translate theoretical knowledge into practice.

Given the single-institution context, the findings are not intended to be broadly generalisable but provide contextually grounded insights. Future research across multiple institutions, as well as studies incorporating student perspectives, could deepen understanding of assessment design practices. Further exploration of digital tools and enhanced moderation processes may also contribute to addressing identified challenges and supporting the ongoing development of assessment quality in nursing education.
